# Double-blind, randomized, placebo-controlled study to evaluate erenumab-specific central effects: an fMRI study

**DOI:** 10.1186/s10194-023-01709-8

**Published:** 2024-01-09

**Authors:** Hauke Basedau, Kuan-Po Peng, Marlene Schellong, Arne May

**Affiliations:** Department of Systems Neuroscience, University Medical Center Hamburg-Eppendorf Martinistr. 52, 20246 Hamburg, Germany

**Keywords:** Headache, CGRP antibodies, Placebo-effect, fMRI, Hypothalamus, Double-blind

## Abstract

**Objective:**

Given the findings of central effects of erenumab in the literature, we aimed to conduct a rigorous placebo-controlled, double-blind, randomized study to elucidate whether the observed changes are directly attributable to the drug.

**Methods:**

We recruited 44 patients with migraine, randomly assigning them to either the erenumab 70 mg or the placebo group. 40 patients underwent fMRI scanning using a trigeminal nociceptive paradigm both, pre- and four weeks post-treatment. Participants kept a headache diary throughout the whole study period of two months in total. A clinical response was defined as a ≥30% reduction in headache frequency at follow-up. Details of this study have been preregistered in the open science framework: https://osf.io/ygf3t.

**Results:**

Seven participants of the verum group (*n=*33.33%) and 4 of the placebo group (21.05%) experienced improvements in migraine activity, characterized by a minimum of 30% reduction in monthly headache frequency compared to baseline. The imaging data show an interaction between the verum medication and the response. Whilst numbers were too small for individual analyses (Verum vs. Placebo and Responder vs. Non-Responder), the variance-weighted analysis (Verum vs Placebo, scan before vs after weighted for response) revealed specific decrease in thalamic, opercular and putamen activity.

**Interpretation:**

The central effects of erenumab could be reproduced in a placebo randomized design, further confirming its central role in migraine modulation. The mechanism, whether direct or secondary to peripheral mode of action, needs further exploration. It is important to note that the response rate to erenumab 70mg in this study was not as substantial as anticipated in 2019, when this study was planned. This resulted in a too small sample size for a subgroup analysis based on the responder status was associated with both the verum drug and the relative reduction in headache days.

## Introduction

Migraine, originally interpreted as a vascular disorder, has undergone a profound evolution in our understanding of its pathophysiological background over the past century [1, 2]. Historically, extracerebral (meningeal) vessels were implicated as the primary drivers of the head pain in migraine. However, modern research suggests that the central nervous system, particularly the hypothalamus, midbrain and brainstem structures play important roles in migraine attack generation [3–6]. This shift in understanding coincided with the advent of a new generation of migraine-specific therapeutics: the monoclonal antibodies targeting the calcitonin gene-related peptide (CGRP) and its receptor (CGRP-mAb). These new treatments emerge from the recognized role of CGRP in migraine pain. CGRP receptors are abundant in the human body and are also found in brain regions which play a pivotal role in the pathophysiology of migraine [6–9].

However, an intriguing paradox exists. Whilst CGRP-mAbs might seem like the ideal candidates to act within the CNS given their central molecular targets, they only marginally traverse the blood-brain barrier (BBB) [10, 11] suggesting that the primary site of CGRP-mAb probably lies outside the BBB [12]. Yet, given the preventive nature of CGRP-mAb effects, notably the reduction in migraine days [13–15], the question arises how these antibodies, despite their limited access to the CNS, influence the genesis and perpetuation of migraine attacks [16, 17]. Using high-resolution, event-related BOLD brainstem functional imaging in migraine patients before and after administration of erenumab [5] and galcanezumab [18], demonstrated that the treatment with CGRP antibodies is associated with a change of brain processing of trigeminal nociceptive input. Moreover, treatment responders in both studies showed more activation in migraine attack generating CNS structures, namely the hypothalamus [5, 18]. Recently, studies showed that Fremanezumab can be found in the CSF and therefore crosses the blood-brain barrier, and next to neuroimaging [5, 18], electrophysiological studies [17, 19, 20] also strongly suggested that CGRP antibodies exhibit robust central effects. None of the mentioned studies tested these findings against placebo. We therefore conducted a placebo-controlled, double-blind, randomized study to elucidate whether 70mg of Erenumab exhibits central effects.

## Material and methods

### Study design

This study was preregistered on May 04, 2020 via the Open Science Framework (https://osf.io/ygf3t). Ethical approval was granted by the Hamburg ethics committee (PV 5964), and all procedures adhered to the Declaration of Helsinki. Informed written consent was secured before initiating study sessions.

### Participants

Migraine patients were recruited from the headache outpatient clinic of the University Medical Center Hamburg. Eligibility was based on (i) ICHD-3 migraine diagnostic criteria [21], (ii) being scheduled for erenumab 70mg treatment per national guidelines [22], and (iii) no prior CGRP-antibody treatment. Stable preventive or other treatments for the last 3 months which were not changed during the study phase were permitted, and primary or secondary headache comorbidities as well as severe other comorbidities were excluded. Patients with medication overuse headache were excluded.

### Experimental setup

Participants underwent two fMRI sessions: Visit 1 was pre and Visit 2 post erenumab administration, with a four-week inter-session interval aligning with medication administration interval (double-blinded administration of a total of two cycles). After the initial scan, 70 mg erenumab or mass equivalent dose sodium chloride 0.9% solution was subcutaneously administered using identically looking syringes by a medical person (MS) not involved in patient recruitment, data acquisition or data analyses. The experimental paradigm [5, 18, 23, 24] consisted of various sensory stimulations, including painful trigeminal stimuli, with subsequent participant ratings. Headache diaries were compiled across the whole study duration and one additional month until unblinding (Fig. 2A).

### MRI protocol

Imaging was executed on a Siemens PRISMA 3T MR system (Siemens, Erlangen, Germany) using a 64-channel head coil. Functional imaging parameters were optimized for BOLD brainstem imaging [9]: voxel size 1,3 x 1,3 x 2,5 mm^3^, 38 axial slices (no gap), repetition time 2.64 s, echo time 28 ms, flip angle 80°, GRAPPA acceleration mode, field of view readout 216 mm, phase partial Fourier 7/8, two saturation pulses were added anterior and posterior to the target volume, which covered the whole volume from the corpus callosum to the foramen magnum (MNI z-range 25 to -72). Simultaneously, we recorded pulse and breathing (Expression, Philipps, Best, Netherlands) to correct for cardiovascular artifacts.

Functional imaging was followed by field mapping MRI sequence (repetition time 0,8 s, echo time 1: 5.51 ms, echo time 2: 7.97 ms, flip angle 40°, field of view readout 215 mm) and a high-resolution magnetization-prepared rapid gradient echo sequence (MPRAGE) image (voxel size 1 mm^3^, repetition time 2.3 s, echo time 2.98 ms, flip angle 9°, field of view 256 mm2, 240 axial slices gap 50%).

### Data preprocessing

Preprocessing, conducted via SPM12 (Wellcome Trust Center for Neuroimaging, London, UK), comprised realignment, unwarping, co-registration, and normalization. A 4 mm^3^ Gaussian kernel was utilized for smoothing.

### Statistical approach

A General Linear Model (GLM) analysis was used within each participant providing β-estimates which were used for group statistical analysis. These β values were calculated for each voxel and signify the condition-specific neuronal activity. Therefore, we were using a hemodynamic response function (HRF) to model all four stimulus conditions (ammonia, rose, air puff, visual) and three confound conditions (key press/assessment, attention task, anticipation phase) by convolving their onsets and durations and applying them as regressors in the GLM. For further correction of movements that were not intercepted by the realignment processing, we included the 6 movement regressors provided in the realign and unwarp step mentioned earlier. For physiological noise correction, we included an additional 18 to 20 regressors extracted from each participants' breath and pulse signals using the approach described by Deckers and colleagues [25]. For the main effect the results were corrected for multiple comparisons (family-wise error corrected, *p <* 0.05), for the sub-analysis with a strong *a priori* hypothesis (see preregistration and [26]) we calculated paired and independent one-sided t-test as implemented in the SPM toolbox and used an uncorrected statistical threshold of *p <* 0.001.

### Arterial spin labelling

Considering the potential of erenumab to modulate vasoactivity, ASL was recorded to exclude that any BOLD changes were due to general changes in cerebral blood flow (CBF). The ASL sequence used pulsed ASL (PASL) recorded with 91 repetitions in 17 slices with a TR of 2.6 s (TE 12 ms, 90° flip angle, bolus duration 1800 ms, inversion time 700 ms, PICORE Q2T perfusion mode, voxel size 2 mm x 2mm x 5 mm). The relative CBF maps calculated by the scanner software were co-registered to the anatomical image, warped into MNI space using the transformation calculated on the anatomical image, and smoothed using a 12 mm isotropic Gaussian kernel again using SPM12 (Wellcome Trust Center for Neuroimaging, London, UK).

## MRI effect analysis

Effects associated with ammonia stimulation were examined by pooling data from both visits (main effect). For estimating the impact of erenumab on trigeminal stimulation, first-level contrasts([ammonia stimulation]_visit 1_ vs [ammonia stimulation]_visit 2_) were used for group comparison: Verum vs. Placebo, voxel-wise One-Sample t-Test with group-wise z-scored relative reduction in monthly headache days, using an uncorrected threshold of *p <* 0.001 owing to a strong a priori hypothesis [5], see preregistration.

## Results

### Patient characteristics

**Fig. 1:**
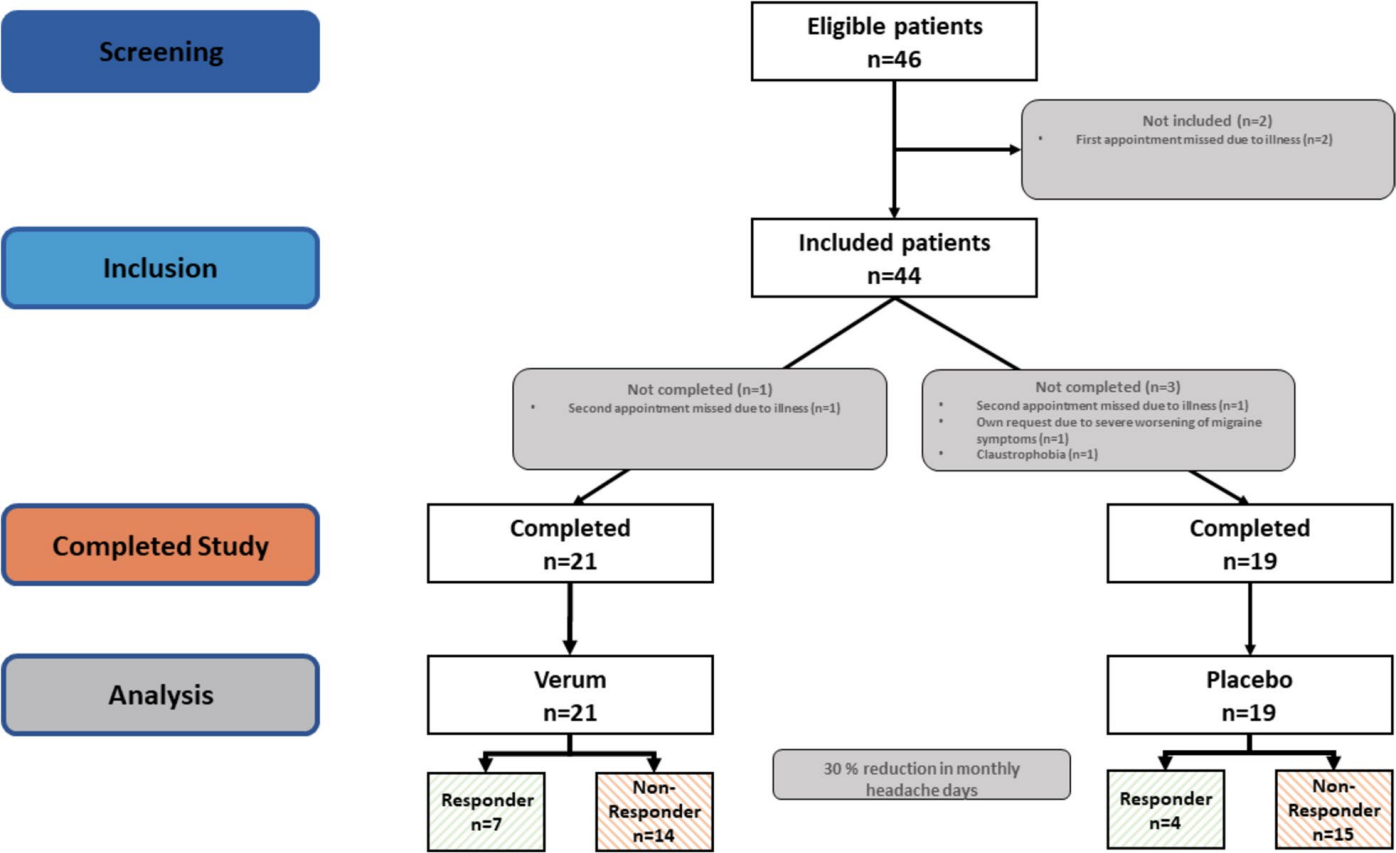
Flow Chart. Flowchart of participant numbers at different study stages and exclusions before and after double-blind randomization

The study encompassed a total of 40 participants, 21 in the Verum group and 19 in the Placebo group. Two participants discontinued the study because of the dosing interval could not be adhered due to illness, another participant discontinued on own request because of severe worsening of migraine symptoms, and another one because of claustrophobia (see Fig. 1). The mean age of participants in the Verum group was 39.1 years (SD = 12.77; range 19-62), and in the Placebo group 41.58 years (SD = 11.43; range 22-60). No significant age difference was observed between groups (*p =* 0.5202). The gender distribution showed that 90.48% (*n =* 19) in the Verum group were female, and 78.95% (*n =* 15) in the Placebo group. The difference was not statistically significant (*p =* 0.398). For overview see Table 1.

**Table 1:** Patient characteristics

***Patient characteristics***	***Verum***	***Placebo***	***Statistics***
**Number**	21	19	
**Female, % (n)**	90.48 (19)	78.95 (15)	^a^0.3976
**Age, mean ± SD (range), in years**	39.1 ± 12,77 (19-62)	41.58 ± 11,43 (22-60)	^b^0.5202
**Migraine with and without aura, n**	8	10	^a^0.5254
**Migraine without aura, n**	**13**	**9**	
**Chronic migraine (ICHD-3), % (n)**	42.86 (9)	21.05 (4)	^a^0.1861
**Episodic migraine (ICHD-3), % (n)**	57.14 (12)	78.95 (15)	
**Baseline headache frequency, mean± SD (range), days/month**	15.68 ± 8.29 (4-30)	13.26 ± 6.51 (6-28)	^c^0.3775
**Absolute reduction in monthly headache frequency after month two mean± SD (range), days/month**	1.69 ± 3.88 (-6-9)	0.98 ± 3.49 (-6-11)	^b^0.5302
**Responder 30% / 50%, % (n)**	33.33 (7) / 19.05 (4)	21.05 (4) / 5.26 (1)	^a^1.00

*Abbreviation ICHD-3* International Classification of Headache Disorders, 3rd edition

^a^Fisher's Exact Test

^b^Two Sample t-test

^c^Wilcoxon rank sum test with continuity correction

### Migraine characteristics

#### Migraine diagnosis

Eight patients in the Verum group (total 21) had a migraine with aura and the same hold true for 10 patients in the Placebo group (total 19). This distribution across groups was not significant (*p =* 0.525). Chronic migraine was diagnosed in 9 patients (42.8%) in the Verum group and 4 patients (21.5%) in the Placebo group. No significant difference was observed across groups (*p =* 0.186).

#### Headache frequency

Baseline: The Verum group reported a mean baseline headache frequency of 15.68 days/month (SD = 8.29; range 4-30), while the Placebo group had an average of 13.26 days/month (SD = 6.51; range 6-28). The difference was not statistically significant (*p =* 0.377).

Reduction after 2 Months: The average reduction in headache frequency after two months for the Verum group was 1.69 days/month (SD = 3.88; range -6 to 9), compared to 0.98 days/month (SD = 3.49; range -6 to 11) for the Placebo group. This change was not statistically significant (*p =* 0.5302).

#### Response rate

30% Responder Rate: The Verum group had a 30% responder rate of 33.33% (*n =* 7) while the Placebo group was at 21.05% (*n =* 4). 50% Responder Rate: For the Verum group, the 50% responder rate was 19.05% (*n =* 4), whereas the Placebo group had a rate of 5.26% (*n =* 1). This difference was not statistically significant (*p =* 1.00). Based on these results, the number required to treat for a 30% reduction in frequency with Erenumab 70mg in our investigation was 8.14, i.e. one would have to treat 8 patients with Erenumab 70mg to have one responder with a 30% reduction in hedache frequency.

#### Behavior/rating analysis

**Fig. 2:**
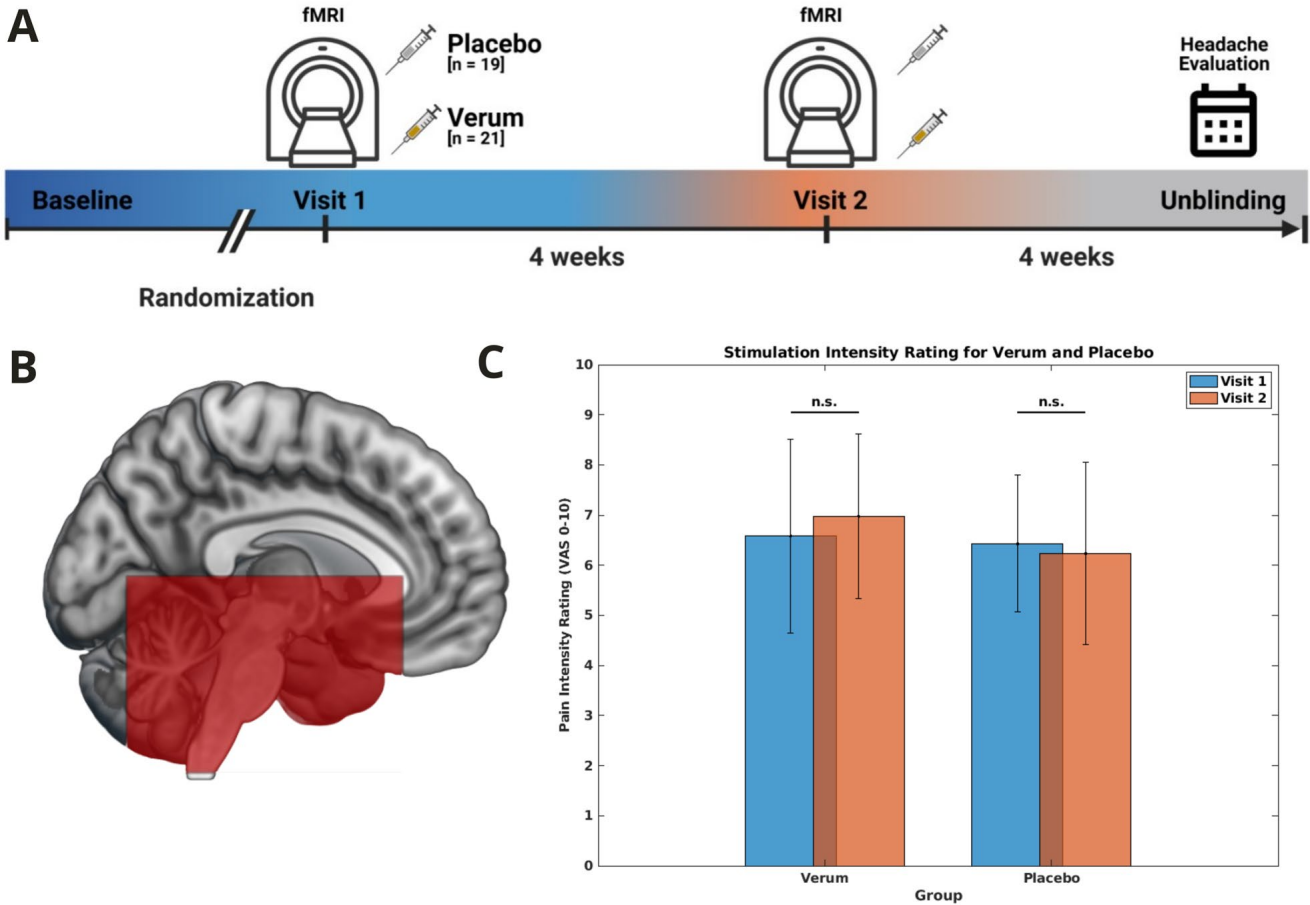
Experimental Design. **A** Experimental design with baseline headache frequency and double-blind randomization to either verum or placebo. **B** Functional Imaging Field of View – EPI Imaging protocol. **C** Behavioral ratings for each group and for both visits for trigeminal nociceptive stimulation on Visual Analogue Scale

Ammonia Pain Intensity Ratings between Visit 1 to Visit 2 (paired sample t-test):

Neither the verum group (t(20) = -1.474, *p =* 0.156) nor the placebo group (t(18) = 0.505, *p =* 0.620; showed a significant difference in ammonia pain intensity ratings between Visit 1 to Visit 2 (paired sample t-test) (Fig. 2C). In the Two-way ANOVA (Verum vs. Placebo) again

neither the Group effect (F(1, 77) = 0.092, *p =* 0.762) nor the Day effect (F(1, 77) = 1.375, *p =* 0.245) were significant.

#### Arterial spin labeling

ASL was performed in a two-step whole-brain protocol and showed no changes in relative CBF (uncorrected, *p <* 0.001, T > 3.32, df = 38).

#### Functional imaging

### Effect of trigeminal stimulation

**Fig. 3:**
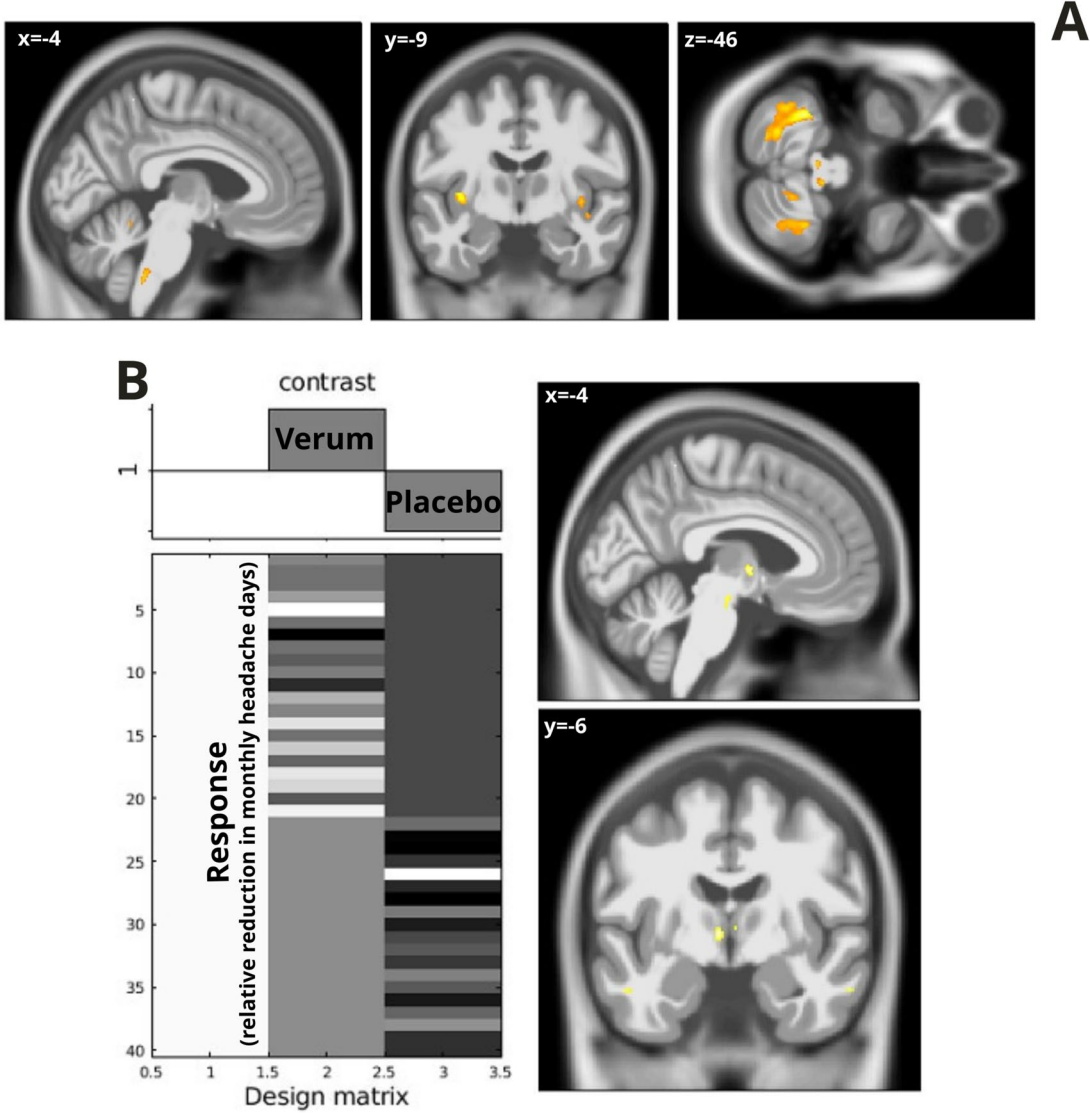
**A** Pooled main effect of trigeminal nociceptive stimulation (visit 1 + visit 2, both groups together). Data is shown at statistical threshold of *p<*0.0001 and a minimum cluster size of 25 voxel and superimposed on the group template. **B** One-Sample t-Test comparing for First-Level contrasts of [ammonia-air puffsVisit1- ammonia-air puffsVisit2]. Additional weighted regressor group-wise z-scored relative change in monthly headache days. Left side: Functional Imaging Design matrix

The cumulative main effect of trigeminal nociceptive stimulation unveiled the involvement of both cortical and subcortical structures that mediate central pain/salience processing. These structures include the ipsilateral/contralateral Spinal Trigeminal Nucleus (STN), the contralateral thalamus, insula, and cerebellum (to the extent included in the Field of View – Fig. 2B). These findings were statistically significant with a threshold of *p<*0.001 (uncorrected) (Fig. 3A, Table 2). Taking Ammonia Pain Intensity Ratings as a confound did not change these results.

**Table 2:** Details of the statistical results of the fMRI analyses

**Anatomical region**	**Cluster size** **(voxels), n**	**T value**	**MNI coordinates** **(x,y,z)**
A) ***Main Effect: Pooled visits, all participants (n = 40),*** ***threshold: p < 0.001 [uncorrected], T > 3.31, minimum, cluster extent 25 voxels, df = 39***			
**L cerebellum exterior**	4501	7.21	-32,-56,-32
**R central operculum**	3139	7.17	50,2,3
**L cerebellum exterior**	3156	6.79	-25,-65,-49
**R cerebellum exterior**	1628	6.15	36,-52,-29
**L posterior insula**	329	5.33	-36,-10,4
**L anterior insula**	121	5.13	-35,5,4
**Cerebellar vermal lobules I-V**	417	5.04	0,-49,-15
**R cerebellum exterior**	765	4.93	32,-56,-49
**R posterior insula**	115	4.42	40,-14,-5
**L brainstem/ spinal trigeminal nucleus**	127	4.41	-3,-42,-49
**R brainstem/ spinal trigeminal nucleus**	122	4.41	7,-39,-47
**L cerebellum exterior**	111	4.38	-13,-44,-22
**R thalamus**	65	4.18	6,-28,-2
**Cerebellar vermal lobules XIII-X**	146	4.15	0,-61,-35
**R cerebellum exterior**	158	4.12	16,-57,-47
**R posterior insula**	27	4.09	41,-1,-16
**R cerebellum exterior**	76	3.94	21,-64,-16
**R putamen**	86	3.92	24,0,-12
**R anterior insula**	30	3.92	38,7,-16
**L central operculum**	96	3.87	-50,6,3
**R putamen**	29	3.87	30,-21,1
**Cerebellar vermal lobules I-V**	72	3.79	-11,-59,-16
**L posterior insula**	35	3.79	-39,-15,-6
**R pallidum**	27	3.63	20,3,1
B) ***Response And Group Variance (n = 40)*** ***contrast [ammonia–air puffs]***_***visit1***_*** > [ammonia–air puffs]***_***visit2***_***,*** ***Verum Response>Placebo Response,*** ***threshold p < 0.001 [uncorrected], T > 3.32, minimum cluster extent 20voxels, df = 37)***			
**R central operculum**	43	4.19	56,8,0
**L putamen**	110	3.91	-20,16,0
**L parahippocampal gyrus**	20	3.78	-4,-6,1
**R putamen**	24	3.74	25,10,-4
**L putamen**	37	3.69	-26,6,-6

A) Main findings of trigeminal pain processing in all participants with both visits pooled. The imaging results are proofing trigeminal nociceptive stimulation and showing typical areas involved in pain processing. B) One-Sample t-Test (*n=*40) with regressor weighting for group (Verum>Placebo) including the Response (relative reduction in monthly headache days). Main findings specific for being a verum responder

### Difference verum and placebo

One-Sample t-Test comparing the First-Level contrasts of [ammonia-air puffs_Visit1-_ ammonia-air puffs_Visit2_] was conducted with additional weighted regressors to differentiate between the Verum and Placebo groups and to consider the group-wise z-scored relative change in monthly headache days during the treatment period.

The results showed a specific decrease in activity within the central Operculum, Putamen, and parahippocampal gyrus. In other words, for individuals in the Verum group who were more likely to respond to the treatment, there was a noticeable reduction in the activity of these areas in response to trigeminal nociceptive pain across the two visits (refer to Table 2B for detailed results, Fig. 3B). Controlling for all clinical relavant factors such as sex, headache side baseline characteristics, and ammonia pain ratings as a covariate into the analysis did not change the results.

## Discussion

The main finding of our study is a decrease of activity in distinct regions within the migraine brain exclusively after the administration of 70mg of erenumab. This alteration in neural activity was particularly observed, and probably mainly driven, by individuals who showed a clinical benefit from the anti CGRP treatment.

However, the pursuit of a wider range of (sub-) analyses did not yield substantial findings, primarily due to the limited number of participants involved in the study and the low response rate of the study cohort. The responsiveness to 70mg of erenumab was notably lower than anticipated during the initial planning phases of the study. When this study was planned in 2019, there were two medications available on the market, with Erenumab emerging as the market leader, and the preferred dosage in daily practice being 70mg. Furthermore, when the study started in 2020 the corona pandemic began and hampered patient recruitment and scanning. After the pandemic subsided and enrolment started again, there were 3 CGRP antibodies available with a 4^th^ coming up, and it became also evident that 140mg of Erenumab were the preferred clinical choice because of higher treatment effects. When the interim clinical analysis revealed a Number Needed to Treat (NNT) exceeding 8, i.e. that we would have needed to study 300 participants for a significant clinical response difference between groups, coupled with correspondingly low case numbers for a sub-cohort analysis, the decision was made to prematurely end the study which was initially planned to enroll 50 patients per group.

Despite the early termination, our data suggest that the variance in trigeminal activity can be exclusively elucidated through a combination of the verum medication and its response interaction. This implies that erenumab induces a central effect, but this alteration is only apparent when the prophylaxis is therapeutically efficient.In our earlier open-label imaging study, erenumab also modulated central pain transmission [5]. Whether these central modulating effects are due to secondary changes after peripheral modulation of sensory input or indeed represents a direct central mode of action needs to be discussed.

The findings suggest a nuanced interplay between erenumab and the central nervous system, underscoring the specificity of the drug’s action in congruence with the individual’s responsive mechanism. This specificity and the observed variance in trigeminal activity may hold significant implications for understanding the mechanistic action of erenumab and its role in modulating neural pathways associated with pain perception and response.

Limitations: Given that the migraine phase can affect the BOLD signal [27], one could argue that since exclusively scanning interictally was not feasible, due to adherence to the exact dosing interval of Erenumab, the migraine phases could explain part of our findings. A total of 20 participants were ictal on day 1 and 16 participants on day 2. In total, 23 of the 44 participants were in the same migraine period on both days: ictal/ictal or interictal/interictal. However, we corrected for the migraine period in the imaging data and, just as in two preceding cohorts [5, 28] could not find any different results in the main effect. We note, that the limitations posed by the sample size and the responsiveness to the intervention necessitate cautious interpretation of the findings and highlight the need for further research with larger cohorts and refined methodologies to validate and expand upon the initial insights gleaned from this study.

## Data Availability

Researchers meeting the criteria for access to confidential data may access anonymized raw data upon reasonable request, involving the documentation of data access.
